# Rising co-payments and continuity of healthcare for Dutch patients with bipolar disorder: retrospective longitudinal cohort study

**DOI:** 10.1192/bjo.2021.994

**Published:** 2021-08-31

**Authors:** Arnold P. M. van der Lee, Ralph Kupka, Lieuwe de Haan, Aartjan T. F. Beekman

**Affiliations:** Department of Psychiatry, VU University Medical Center, Amsterdam University Medical Center, the Netherlands; Department of Psychiatry, VU University Medical Center, Amsterdam University Medical Center, the Netherlands; Department of Psychiatry, Academic Medical Center, Amsterdam University Medical Center, the Netherlands; Department of Psychiatry, VU University Medical Center, Amsterdam University Medical Center, the Netherlands

**Keywords:** Bipolar disorder, co-payments, continuity of care, registry data, the Netherlands

## Abstract

**Background:**

The Netherlands has few financial barriers to access mental healthcare. However, in 2012, a sharp rise in co-payments was introduced.

**Aims:**

We tested whether these increased co-payments coincided with less guideline-recommended continuous out-patient psychiatric care and more crisis interventions for patients with bipolar disorder.

**Method:**

A retrospective longitudinal cohort study on a health insurance registry was performed to examine trends, and deviations from these trends, in the healthcare received by patients with bipolar disorder. Deviations of trends were tested by time-series analyses (autoregressive integrated moving average). Subsequently, the relationship between significant deviations of trends and rise in co-payments was examined. Outcome measures were the level of standard out-patient care (out-patient psychiatric care and/or medication), crisis psychiatric care and somatic care.

**Results:**

The cohort comprised 3210 patients. During follow-up, the use of psychiatric care decreased and somatic care increased. The high rise in co-payments from 2012 onward coincided with decreases in standard out-patient care and increases in medication-only treatment, crisis psychiatric care and somatic care. Crisis intervention was highest when patients received only bipolar disorder medication. Patients receiving continuous standard out-patient care (62%) had less crisis intervention compared with the other patients.

**Conclusions:**

Our data suggest that the rise of co-payments decreased guideline-recommended continuous out-patient psychiatric care among patients with bipolar disorder, and increased crisis psychiatric care.

Bipolar disorder is a chronic and recurrent mental illness that has a considerable impact on emotional, psychosocial and occupational functioning, quality of life, somatic health and life expectancy.^1–4^ Patients need continuous healthcare as recommended by the professional guidelines for bipolar disorder.^5^ The intensity of treatment depends on the phase of the disorder: acute treatment for mania or depression, followed by continuous out-patient maintenance treatment to prevent recurrences and improve well-being and functioning.^6–10^ Use of medication to treat or prevent episodes is ideally embedded in a framework of supportive and psychological treatment. In most patients, this will considerably lower the burden of the illness, even if symptomatic and functional recovery is incomplete in some. This standard out-patient care is typically delivered by mental health institutions, or in primary care for patients who have been stable for several years without the need for complex pharmacotherapy. Crisis intervention is indicated in severe manic or depressive episodes, and consists of intensive out-patient crisis intervention or hospital admission. Patients who decide to stop standard out-patient care and/or medication prophylaxis are at increased risk for recurrence of episodes necessitating crisis care.

## Dutch healthcare system and rising co-payments

In the Netherlands, the healthcare system for patients with bipolar disorder and other severe mental illnesses is well developed.^11–14^ For example, 73% of patients with schizophrenia received continuous care in 2009–2011.^15^ As in many countries, costs of healthcare are rising, resulting in a re-evaluation of how healthcare is financed.^11,16,17^ All inhabitants of the Netherlands have access to good-quality healthcare for somatic and mental health problems, with few financial barriers. Although co-payments are low compared with many other countries, they are rising.^18^ In 2012 and 2013, co-payments per year for all healthcare were raised from €155 to €360. On top of that, during 2012 an additional co-payment only for specialised mental healthcare was charged to stimulate a shift to primary care.^19,20^ Although there is evidence that effects of co-payments may be limited among patients with severe somatic health problems, among patients with mental disorders these effects are larger.^21,22^ The assumption that patients with more severe illnesses will choose to continue using care that they know is of value, the moral hazard hypothesis, seems less applicable for a substantial proportion of patients with bipolar disorder.^23,24^ Co-payments may disrupt the continuity of standard out-patient care that is guideline-recommended for patients with severe mental disorders, and as a consequence, may lead to a rise in the use of acute psychiatric crisis intervention.

## Aim

A natural experiment to study the effects of co-payments on healthcare use of patients with bipolar disorder occurred when the co-payments in the Netherlands were substantially raised in 2012. Overall, use of mental healthcare was substantially lower after this high rise in co-payments, especially among patients with lower incomes.^25^ Some patients with bipolar disorder may be even more vulnerable to unwanted effects of co-payments because of a lower than average income,^21,22^ or limited insight in the recurrent nature of their illness.

Our hypotheses were that over the follow-up period, there would be a trend to diminished use of psychiatric care; that relative to this overall trend, the high rise in co-payments in 2012 would be associated with a further decrease of standard out-patient care and an increase in crisis psychiatric care (in-patient or intensive out-patient crisis treatment); and that patients with bipolar disorder who receive continuous standard out-patient care would use less crisis psychiatric care.

## Method

### Study design and patient selection

All patients with a diagnosis of bipolar disorder in 2008, under 70 years of age on 1 January 2008, and insured by Zilveren Kruis from 2008 to 2014, were selected and analysed in a retrospective cohort study. Patients aged 70 years or older were not included because they may have developed cognitive impairment and more somatic comorbidities, and may therefore have responded differently to co-payments compared with younger patients.^26,27^ Selection and analysis were guided by the strict rules of the Dutch privacy laws and regulations. As the data in the study database could not be linked to individual patients, no informed consent or permission from a medical ethical committee were necessary under Dutch and European laws. A period with few co-payments (2009–2011) was followed by a period (2012–2014) with a high rise in general co-payments and a co-payment for specialised mental healthcare (applied only in 2012). Using this design, trends in care and deviations of these trends in relation to the extent of co-payments could be analysed. Zilveren Kruis is the largest Dutch health insurer and provided the computerised registry data. Zilveren Kruis provided health coverage for about 30% of the 16.4 million residents in the Netherlands in 2008. Those insured by Zilveren Kruis were representative of the Dutch population, according to analyses by Zilveren Kruis. Zilveren Kruis compared their data with national data.^28,29^ All mental and somatic healthcare use, including prescription data of medication for bipolar disorder, of these patients over 2009–2014 were analysed.

### Data source: Dutch computerised health insurance registry data

All data were extracted from the Zilveren Kruis health insurance registry database, which contains all care under the Dutch Health Insurance Law. Dutch health insurance companies have to follow the strict regulations by the National Care Authority. The Zilveren Kruis database contains all of the care patients received from all of their healthcare providers.

Healthcare providers are paid depending on the diagnosis (diagnosis treatment combination, in Dutch language: *diagnose behandel combinatie*, abbreviated as DBC). The diagnostic information on DBC claims is limited. The main groups of diagnoses of DSM-IV are registered on DBC claims, not the detailed codes (fourth and fifth digit) of the specific phases of bipolar disorder. No diagnosis has to be provided for short-term treatment. If more than one psychiatric disorder is treated at the same time, separate DBC claims are paid. Treatment for a psychiatric crisis situation is specified on the DBC claim. The maximum duration for DBC claims is 1 year, except for crisis treatment, for which the maximum duration is 28 days. There are separate tariffs for out-patient care (per minute) and in-patient care (per day and accounting for intensity of treatment). Data for out-patient medication included the defined daily dose (DDD).^30^ There was no information on in-patient medication in the data-set.

### Co-payments

In the Netherlands, co-payments were low compared with other countries, but the general co-payments for all care had risen from €155 in 2009 to €165 in 2010, €170 in 2011, €220 in 2012, €350 in 2013 and €360 in 2014.

Applied only in 2012, extra specific co-payments for psychiatric care had to be paid by patients of 18 years and older. These co-payments were as follows: for out-patient psychiatric care, €100–€200 per year, and for in-patient care, €145 per month starting at the second month of treatment. Assertive community treatment, compulsory hospital stays and crisis treatment were excluded from these co-payments.^19^ The height of co-payments per individual patient are not available in our data-set.

### Measures: cross-sectional per quarter of each year

All healthcare delivered under the Health Insurance Law^11,12^ was divided into standard out-patient care (non-crisis psychiatric out-patient care with or without medication for bipolar disorder, as recommended in the treatment guideline for bipolar disorder), crisis psychiatric care (out-patient psychiatric crisis treatment or in-patient psychiatric care) and somatic care (all other healthcare). Because a treatment episode in the Dutch DBC claim system can last up to 365 days, every quarter contained patients who continued a treatment, started a new treatment or had no treatment at all. In each quarter, a patient could have any combination of standard out-patient care, crisis psychiatric care or somatic care.

Medication for bipolar disorder consists of lithium, anticonvulsants, antipsychotics or antidepressants. These medications are used for the acute treatment of manic and depressive episodes, and maintenance treatment to prevent recurrences of bipolar disorder. Medication was attributed to the quarter in which it was collected from the pharmacy, and the amount was measured in DDD.

We also counted the number of patients starting a new standard out-patient care DBC or a new crisis psychiatric care DBC per quarter. The average amount of psychiatric care per patient per quarter as reflected by the costs of care was calculated with average national prices in Euros, and allocated to the quarter of the initial date. The costs were adjusted for inflation, using price indices for psychiatric care based on the prices of 2009.^31,32^

The amount of somatic care (all non-psychiatric care including all medication for other conditions than bipolar disorder) was calculated as the sum of costs of somatic care per quarter. Somatic treatment was attributed to the quarter of the starting day of the somatic treatment. The amount of somatic care was adjusted with cost indices for somatic care calculated over all insured by Zilveren Kruis, using the prices of 2009 as a basis. Continuity of standard out-patient care was defined as receiving standard out-patient care in every quarter of 2009–2014.

All measures were calculated per quarter of the consecutive years 2009–2014.

### Analysis

The aim of our analysis was to examine whether changes in standard out-patient care, crisis psychiatric care and somatic care were related to the substantial rise in co-payments from 2012 onward. To do so, we analysed trends in healthcare use over 2009–2014 and examined deviations from these trends using data per quarter of the year.

Data per quarter of the year can show repeating seasonal changes; for example, the first and second quarters of each year show lower levels of care usage compared with the third and fourth quarters. In such cases, time-series analyses is the statistical method to examine data that show trends and seasonal patterns, and statistically test deviations of these trends and patterns.^33^ Deviations in the trends of healthcare usage over 2009–2014 were statistically tested for significant differences by time-series analyses, applying Box-Jenkins autoregressive integrated moving average (ARIMA) models^34,35^ (for more explanation please see Methods in the Supplementary material available at https://doi.org/10.1192/bjo.2021.994). All analyses were performed with SAS Enterprise guide version 6.1 for Windows (SAS Institute Cary, North Carolina, USA; see https://www.sas.com/en_us/home.html).

## Results

**Fig. 1:**
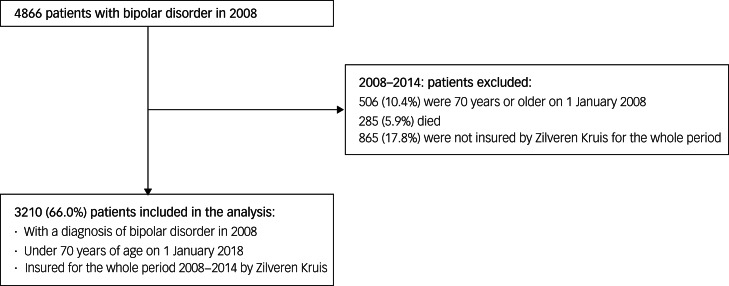
Flow chart of inclusion of 3210 patients with bipolar disorder.

The cohort included 3210 patients with a diagnosis of bipolar disorder in 2008, who were under 70 years of age on 1 January 2008, and were insured by Zilveren Kruis from 2008 to 2014 (Fig. 1). Of these, 39% were male (mean age 46.5 years, s.d. 12.7) and 61% were female (mean age 46.8 years, s.d. 12.7), comparable to the national percentages for men (41%) and women (59%).

### Standard out-patient care

**Fig. 2:**
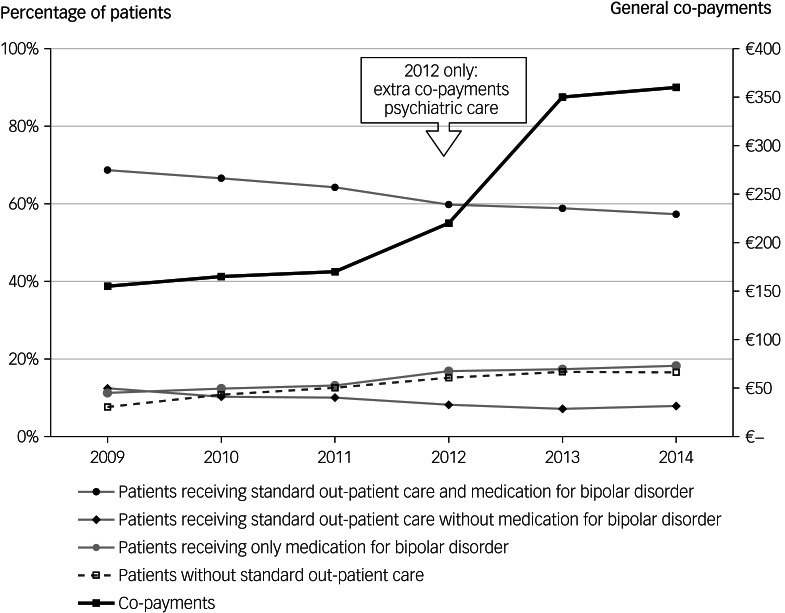
Standard out-patient care in relation to co-payments for psychiatric care.

We assessed changes in standard care per year (Table 1). Over the entire period 2009–2014, the use of standard care decreased. The percentage of patients receiving standard out-patient care with medication for bipolar disorder decreased from 69 to 57% (−17%). Standard out-patient care without bipolar disorder medication decreased from 12 to 8% (−37%). Receiving only bipolar disorder medication increased from 11 to 18% (62%), whereas the percentage of patients without any standard out-patient care more than doubled from 8 to 17% (117%) (Table 1 and Fig. 2). Overall, the percentage of patients receiving standard out-patient care, with or without bipolar disorder medication, declined from 81 to 65% (−20%), and the percentage of patients with medication for bipolar disorder declined from 80 to 76% (−5%). The number of patients starting a new standard out-patient care DBC declined by 24%. The amount of standard out-patient care over all patients in the cohort, measured in Euros, declined by 23%. The amount of medication for bipolar disorder over all patients in the cohort, measured in DDD, declined by 4%. With regard to the type of medication prescribed for bipolar disorder, the use of antipsychotics increased from 44 to 45% (3%), whereas the use of lithium declined from 42 to 37% (−13%) over 2009–2014 (Table 1). The use of anticonvulsants and antidepressants remained constant at around 30%. Over the whole period, 1999 (62%) of the 3210 patients in the cohort accessed continuity of standard out-patient care.

**Table 1:** Trends in psychiatric and somatic care over 2009–2014^a^

	Averages per year				% Change
	2009		2014		2019–2014
	*n*	%	*n*	%	
Standard out-patient care					
Patients receiving standard out-patient care with medication for bipolar disorder	2205	69%	1840	57%	−17%
Patients receiving standard out-patient care without medication for bipolar disorder	400	12%	253	8%	−37%
Patients receiving only medication for bipolar disorder	361	11%	586	18%	62%
Patients without standard out-patient care	245	8%	532	17%	117%
All patients receiving standard out-patient care	2604	81%	2093	65%	−20%
All patients receiving medication for bipolar disorder	2566	80%	2426	76%	−5%
Lithium	1356	42%	1173	37%	−13%
Antipsychotic medication	1397	44%	1438	45%	3%
Anticonvulsant medication	947	30%	958	30%	1%
Antidepressant medication	988	31%	933	29%	−6%
No medication for bipolar disorder	644	20%	784	24%	22%
Number of patients starting a new standard out-patient care DBC	702	22%	531	17%	−24%
Amount of standard out-patient care per patient (average costs in Euros)	583		448		−23%
Amount of medication for bipolar disorder (average number of DDD)	131		126		−4%
Crisis psychiatric care					
All patients	604	19%	388	12%	−36%
Patients receiving standard out-patient care with medication for bipolar disorder	210	10%	148	8%	−15%
Patients receiving standard out-patient care without medication for bipolar disorder	40	10%	24	10%	−5%
Patients receiving only medication for bipolar disorder	280	77%	187	32%	−59%
Patients without standard out-patient care	74	32%	28	5%	−83%
Subgroup with continuity of standard out-patient care^b^	308	15%	204	10%	−34%
Subgroup with quarters without standard out-patient care	296	24%	184	15%	−38%
Number of patients starting a new crisis psychiatric care DBC	175	5%	151	5%	−14%
Amount of crisis psychiatric care per patient (average costs in Euros)	1343		1087		−19%
Somatic care					
Average adjusted costs of somatic care (Euros)	617		884		43%

DBC, *diagnose behandel combinatie* (diagnosis treatment combination); DDD, defined daily dose.

a. Averages over the quarters per year, please see Supplementary material for the data per quarter.

b. Continuity of standard out-patient care was defined as receiving standard out-patient care during every quarter of 2009–2014.

**Fig. 3:**
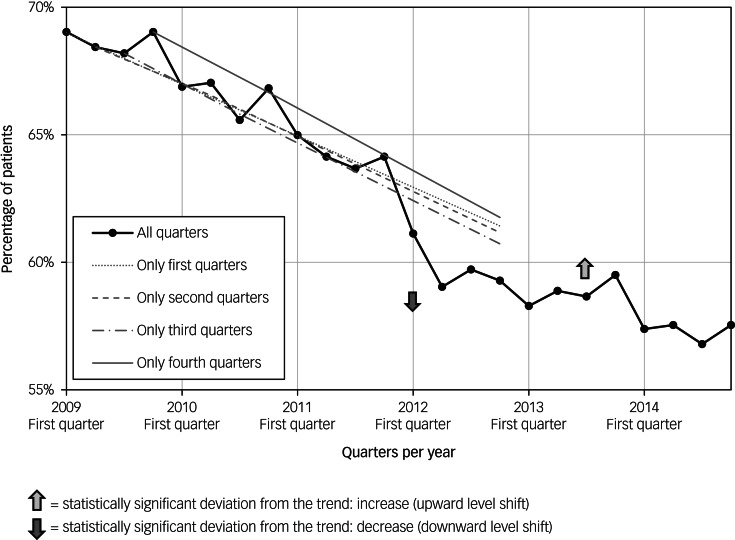
Percentage of patients receiving standard out-patient care with medication for bipolar disorder. Time-series analyses is a two-step process. First, the data are visually inspected; second, based on the observations, a time-series model will be built to statistically test observed deviations. In this example we show this process. First, starting inspection, a decreasing trend can be observed. The trend is not decreasing evenly, but shows a seasonal pattern. For example, the points of the fourth quarters of 2009–2011, are higher than the points of the other quarters in the same year, with an exception in 2012. Next, deviations of this trend and the seasonal pattern are examined. The deviations appear to begin at the first quarter of 2012. This can be seen in Fig. 3 because the point of the first quarter of 2012 is lower than would be expected, which can be seen by drawing a line connecting the points of the first quarters in 2009–2011 and extending the line into 2012. In the same way, the points of the quarters 2, 3 and 4 of 2012 are all lower than would be expected by extending the lines of their corresponding quarters. Therefore, the decreasing trend decreases even more in 2012. In autoregressive integrated moving average (ARIMA) modelling this is called a downward level shift. The second step in the time-series analyses is to test if the observed deviations, downward or upward level shifts, are statistically significant, using ARIMA modelling. The data per quarter of the year can be found in Supplementary Tables 1 and 2 with the statistical results of the time-series analyses.

Next, we examined if the above-mentioned trends in standard out-patient care showed significant deviations, taking into account seasonal patterns (e.g. Fig. 3), and tested any deviations by using time-series analyses (ARIMA).

**Fig. 4:**
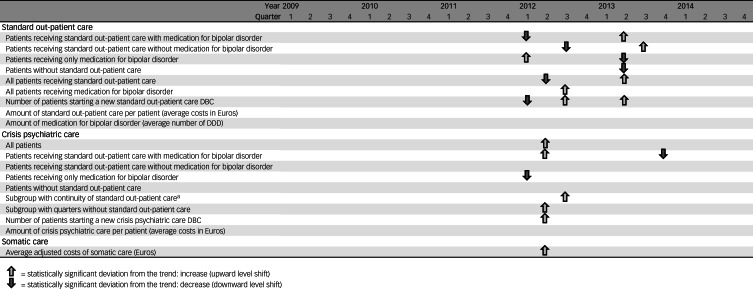
Statistically significant deviations of the general trends in standard out-patient care, crisis psychiatric care and somatic care: increases and decreases. ^a^Continuity of standard out-patient care was defined as receiving standard out-patient care during every quarter of 2009–2014. DBC, *diagnose behandel combinatie* (diagnosis treatment combination); DDD, defined daily dose.

The following statistically significant deviations from the general trends of care consumption were observed (for details, refer to Fig. 4 and Supplementary Tables 1 and 2). First, the decreasing trend of all patients receiving standard out-patient care decreased even more in the second quarter of 2012, and increased to a higher level in the second quarter of 2013. When we divided patients in subgroups based on the type of standard out-patient care received, with or without bipolar disorder medication, we found comparable deviations from the trends. Second, the increasing trends of the use of bipolar disorder medication increased even more in 2012. This was observed in all patients receiving bipolar disorder medication and in those treated with only bipolar disorder medication. Third, the increasing trend of patients without standard out-patient care slowed in the second quarter of 2013.

### Crisis psychiatric care

We assessed the course of crisis psychiatric standard care per year (Table 1). First, over the entire period (2009–2014) the percentage of patients receiving crisis psychiatric care declined from 19 to 12% (−36%) (Table 1). Likewise, the amount of crisis psychiatric care, measured in Euros, also declined (−19%). Second, the level of crisis psychiatric care was related to the type of standard out-patient care received. Patients receiving standard out-patient care, with or without bipolar disorder medication, showed crisis psychiatric care levels of 10% on average per year over 2009–2014. Patients who only received bipolar disorder medication showed very high levels of crisis psychiatric care, 77% received crisis psychiatric care in 2009, declining to 32% in 2014 (−59%). The percentage of those patients with only bipolar disorder medication receiving crisis psychiatric care was 4.1 times the average in 2009, and still 2.6 times more in 2014. Third, in the subgroup of 1999 patients receiving continuity of standard out-patient care, 10% received crisis psychiatric care in 2014 compared with 15% of the other 1211 patients.

Next, we used time-series analyses to examine whether the declining trends in crisis psychiatric care showed significant deviations (for details, refer to Fig. 4 and Supplementary Tables 1 and 2). Over all patients, relative to the decreasing trend in crisis psychiatric care, there was a significant increase in the use of crisis care, which started in the second quarter of 2012. A comparable significant deviation of the trend was also found in most subgroups, except in patients receiving only bipolar disorder medication. There, we observed a significant decrease from the decreasing trend in crisis psychiatric care in the first quarter of 2012. However, the level of crisis psychiatric care (32% in 2014) remained very high in these patients receiving only bipolar medication.

### Somatic care

The average amount of somatic care per patient per year rose considerably by 43% (Table 1), and significantly rose even more from the second quarter of 2012 onward (see Supplementary Tables 1 and 2).

## Discussion

We studied the healthcare received over 2009–2014 by a cohort of patients diagnosed in 2008 with bipolar disorder in relationship to substantially rising co-payments from 2012 onward. Our trend analyses show that the rise in co-payments from 2012 onward indeed coincided with significant deviations of ongoing trends in healthcare use. These deviations amount to a further acceleration of decreases in standard out-patient care, further increases in patients treated with only medication for bipolar disorder, and increases in the use of crisis psychiatric care.

Our most important findings about the trends over this period are that overall psychiatric healthcare, standard out-patient care and crisis psychiatric care decreased strongly. The percentage of patients without any guideline-recommended standard out-patient care more than doubled. The percentage of patients who only used medication for bipolar disorder increased. The use of somatic healthcare increased considerably between 2009 and 2014. Although the overall decreasing trends in use of standard out-patient care is in itself very worrying and warrants exploration, the object of this study was to test the effects that rising co-payments may have had on top of existing trends.

A total of 62% of the patients in this cohort continuously received standard out-patient care. Their level of crisis psychiatric care was two-thirds of the level observed in the other patients. These results suggest that when patients are not receiving continuous standard out-patient care, the risk of occurrence of crisis treatment is elevated. Crisis psychiatric care was highest in quarters when patients received only medication for bipolar disorder, and second highest in quarters when patients received no standard out-patient care at all. This supports the hypothesis that disruption of standard out-patient care may add to severe relapses or recurrences and the use of crisis psychiatric care.

Although causal inferences from a naturalistic study should be treated with caution, our results support the hypothesis that co-payments have unwanted effects on the continuity of guideline-recommended care among patients with bipolar disorder.^23–25^ We observed diminishing levels of standard out-patient care and increasing levels of crisis care concurrent with extra co-payments for specialist psychiatric care. When these co-payments were introduced they were controversial, and several measures were taken to mitigate the anticipated effects. Some care providers refused to collect co-payments from patients, and several cities and health insurance companies arranged contracts for low-income groups and reduced the co-payments. Patients with bipolar disorder and low incomes, in a period of economic recession with even slightly decreasing purchasing power,^36^ may have benefited from these contracts. Without such measures, the effects of co-payments we found may have been even larger.

Given that this is a naturalistic study, we were alert to other influences that may explain our findings. Still, as all patients in our cohort were insured by the same company, they were subject to the same healthcare policies, co-payments and changes in them, and the same procurement policy of one healthcare insurer. In 2012, the high rise in co-payments was the only major change in national healthcare policies.

### Strengths and limitations

Our study of the relationship between co-payments, continuity of guideline-recommended standard care and crisis psychiatric care, and using crisis psychiatric care as a proxy of lower quality of care, offers comparable findings to a previous study on schizophrenia,^25^ and has the following strengths: following a cohort of diagnosed patients with bipolar disorder over a period with few co-payments (2009–2011), followed by a high rise in co-payments (2012–2014); using the complete records of all healthcare they received; and encompassing the complete set of psychiatric and somatic healthcare including medication as delivered by all of the healthcare providers under Dutch law, including psychiatric care provided by psychiatrists working in private practices, and psychiatric departments of general hospitals.

Research using large registry databases complements the insights provided by clinical trials, especially about the effects on large groups in the population.^37^ The few population studies available only analyse one aspect of healthcare for patients with bipolar disorder; for instance the use of medication, costs, family services or continuity of care.^4,10,25,38^ The results of the present study are relevant for countries like the Netherlands planning to raise or to introduce co-payments.

Our study has several limitations. First, this is a naturalistic study open to bias resulting from selection and attrition of participants. Second, other factors than the co-payments may have had an impact on care use in ways unknown to us. Third, the use of healthcare insurance data provides little data on the clinical and demographic characteristics of patients. It is likely that both clinical and environmental factors moderate the effects of co-payments on healthcare use. The strength of our study is evaluating trends of psychiatric treatment and effects of co-payments in a large cohort of patients with a reliable diagnosis of bipolar disorder. However, the available data did not include detailed clinical information about, for instance, comorbid substance use disorders, suicidality or somatic comorbidities, which could be relevant to understand which clinical factors are associated with changes in treatment.^1,2,26,39^ We recommend to explore the reasons why patients were avoiding adequate treatment and why crisis care was increasing. Specifically, the role of suicidality, substance misuse, comorbidities and other patient characteristics on crisis treatment, and on avoiding adequate treatment, should be evaluated. Moreover, the influence of changes in patient costs on these associations should be explored. This would require a new study, using linked patient data from a combination of registry data and clinical and environmental data. Our findings should be seen as overall effects of co-payments indicating that further study of effects of financial incentives is urgently needed.

Our findings support the hypotheses that co-payments have a disruptive effect on the continuity of standard guideline-recommended out-patient care among patients with bipolar disorder, that this disruption adds to the risk of recurrence of acute episodes, and that this leads to increased use of crisis psychiatric care Discrimination of patient with bipolar disorder, who are vulnerable and often have low incomes, by requesting an extra financial burden is contrary to a legitimate healthcare policy.

Since we used observational data, firm causal inferences about the association between the co-payments and healthcare for patients with bipolar disorder cannot be made. However, as it is unlikely that an experimental study testing the effects of co-payments will ever be done, these are probably the best data we have. Given the importance of continuous care for patients with bipolar disorder, the results give reason to avoid the introduction of co-payments.

## Data Availability

The registry data that were used in this study are not publicly available owing to Dutch privacy laws and regulations.

## Supplementary material

For supplementary material accompanying this paper visit http://dx.doi.org/10.1192/bjo.2021.994. click here to view supplementary material
