# Supplementary material for: Rising co-payments and continuity of healthcare for Dutch patients with bipolar disorder: retrospective longitudinal cohort study
Source: BJPsych Open. 2021 Aug 31;7(5):e155. doi: 10.1192/bjo.2021.994 (PMC8444049; doi:10.1192/bjo.2021.994)
Supplement: Supplementary file 1 [file S2056472421009947sup001.pdf]

Supplement:

METHODS: Analysis of deviations of trends with Box-Jenkins autoregressive integrated moving average (ARIMA) models

The aim of the analysis of the trends and deviations of the trends in use of healthcare was to examine our hypothesis that the rise in co-payments coincided with a decrease in elective outpatient psychiatric care and an increase of episodic psychiatric care among patients with bipolar disorder. We first inspected the trends per quarter over the follow-up period 2009-2014 graphically to observe deviations of these trends. In our study examples of deviations of trends are that the trend continues to decrease or increase, but at a higher or lower level (upward and downward level shifts). In other words, a decreasing trend can have a change to a higher level, but still be decreasing after that point in time, see schematic examples in figure 1.
When changes in trends of care usage over time were observed these were tested by Box-Jenkins autoregressive integrated moving average (ARIMA) models.[1–7] These models account for autocorrelation, nonstationarity and seasonal changes in the trends. The care data showed seasonal patterns per quarter over the years, therefore the fourth difference was used to make the time series stationary. To assess the changes in trends we applied the augmented Dickey Fuller (ADF) test and the (partial) autocorrelation functions were used to assess the 'stationarity' of the models. The 'white noise' assumption was assessed by checking if the autocorrelations differ significantly from 0 (Ljung-Box statistic). Goodness of fit was tested with the Akaike Information Criterium (AIC) and Bayesian Information Criterium (BIC). By developing one ARIMA model for each of the time series there was no multiple testing and no corrections for multiple testing were necessary. All analysis was performed with SAS Enterprise guide 6.1 [SAS Institute Cary, NC, USA].

References

1. Box GEP, Jenkins GM. Time series analysis: forecasting and control, revised edition. San Francisco: Holden Day; 1976.

2. SAS Institue Inc. SAS/ETS 9.2 User's Guide [Internet]. Cary, NC, USA; 2008. Available from: http://support.sas.com/documentation/cdl/en/etsug/60372/PDF/default/etsug.pdf

3. Chin KL, Tacey M, Reid CM, Tonkin A, Hopper I, Brennan A, et al. Temporal Changes in Characteristics, Treatment and Outcomes of Heart Failure Patients Undergoing Percutaneous Coronary Intervention Findings From Melbourne Interventional Group Registry. Hear Lung Circ [Internet]. 2019 Jul 1;28(7):1018–26. Available from: https://linkinghub.elsevier.com/retrieve/pii/S1443950618306942

4. Box GEP, Pierce DA. Distribution of Residual in Autoregressive- Autocorrelations Integrated Moving Average Time Series Models. J Am Stat Assoc [Internet]. 1970;65(332):1509–26. Available from: http://www.jstor.org/stable/2284333 .

5. Beard E, Marsden J, Brown J, Tombor I, Stapleton J, Michie S, et al. Understanding and using time series analyses in addiction research. Addiction [Internet]. 2019 Oct;114(10):1866–84. Available from: https://onlinelibrary.wiley.com/doi/abs/10.1111/add.14643

6. Jandoc R, Burden AM, Mamdani M, Lévesque LE, Cadarette SM. Interrupted time series analysis in drug utilization research is increasing: systematic review and recommendations. J Clin Epidemiol [Internet]. 2015 Aug;68(8):950–6. Available from: https://linkinghub.elsevier.com/retrieve/pii/S0895435615001237

7. Helfenstein U. Box-Jenkins modelling in medical research. Stat Methods Med Res [Internet]. 1996 Mar 2;5(1):3–22. Available from: http://journals.sagepub.com/doi/10.1177/096228029600500102

Figure 1 Schematic examples of trends with upward and downward level shifts

Figure 1A Upward trend with upward level shift

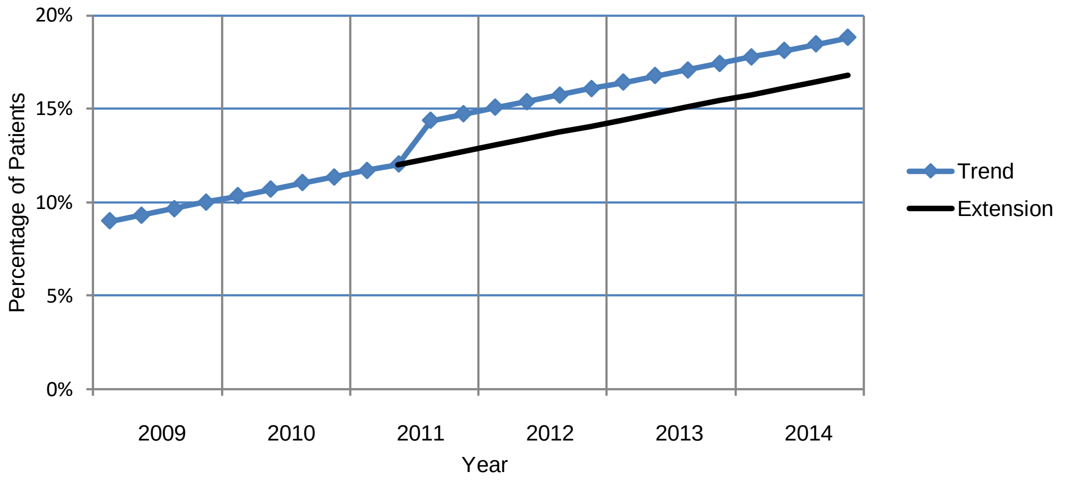

Figure 1B Downward trend with downward and upward level shift

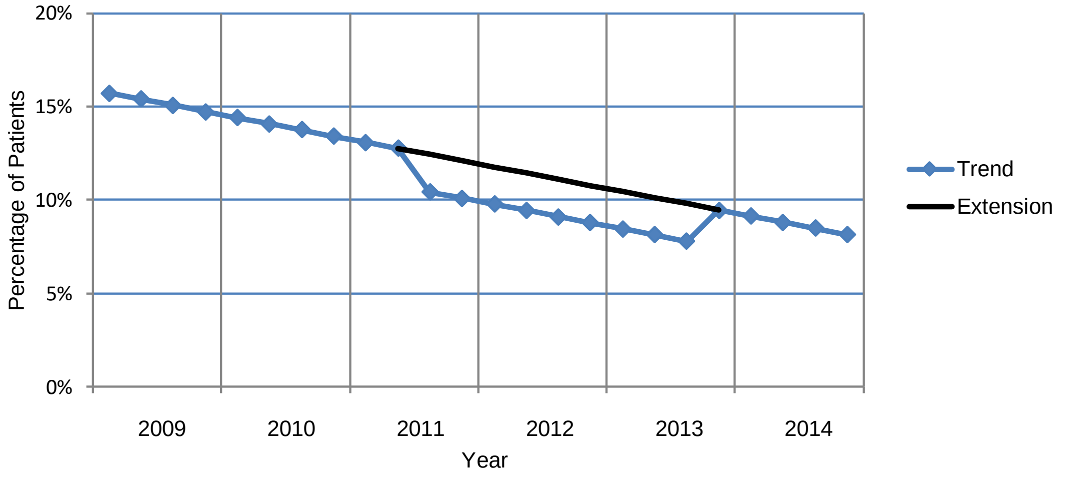

Table 1 Trends of psychiatric and somatic care and general co-payments, results of time series analysis (ARIMA) over 2009-2014

Year	2009								2010								2011							
Quarter	1		2		3		4		1		2		3		4		1		2		3		4	
	N	%	N	%	N	%	N	%	N	%	N	%	N	%	N	%	N	%	N	%	N	%	N	%
**Standard outpatient care**																								
Patients receiving standard outpatient care with medication for BD	2216	69%	2197	68%	2189	68%	2216	69%	2147	67%	2152	67%	2105	66%	2145	67%	2086	65%	2059	64%	2044	64%	2059	64%
Patients receiving standard outpatient care without medication for BD	439	14%	404	13%	379	12%	376	12%	348	11%	324	10%	339	11%	308	10%	329	10%	328	10%	328	10%	306	10%
Patients receiving only medication for BD	361	11%	383	12%	372	12%	329	10%	398	12%	390	12%	404	13%	396	12%	398	12%	440	14%	419	13%	430	13%
Patients without standard outpatient care	194	6%	226	7%	270	8%	289	9%	317	10%	344	11%	362	11%	361	11%	397	12%	383	12%	419	13%	415	13%
All Patients receiving standard outpatient care	2655	83%	2601	81%	2568	80%	2592	81%	2495	78%	2476	77%	2444	76%	2453	76%	2415	75%	2387	74%	2372	74%	2365	74%
All Patients receiving medication for BD	2577	80%	2580	80%	2561	80%	2545	79%	2545	79%	2542	79%	2509	78%	2541	79%	2484	77%	2499	78%	2463	77%	2489	78%
Lithium	1384	43%	1363	42%	1340	42%	1335	42%	1324	41%	1336	42%	1307	41%	1328	41%	1294	40%	1305	41%	1272	40%	1285	40%
Antipsychotic medication	1397	44%	1401	44%	1399	44%	1391	43%	1383	43%	1396	44%	1403	44%	1416	44%	1383	43%	1409	44%	1397	44%	1424	44%
Anticonvulsant medication	943	29%	938	29%	953	30%	955	30%	952	30%	956	30%	942	29%	939	29%	929	29%	932	29%	940	29%	933	29%
Antidepressant medication	999	31%	1015	32%	973	30%	967	30%	971	30%	967	30%	939	29%	951	30%	950	30%	953	30%	947	30%	940	29%
No bipolar disorder medication	633	20%	630	20%	649	20%	665	21%	665	21%	668	21%	701	22%	669	21%	726	23%	711	22%	747	23%	721	22%
Number of patients starting a new standard outpatient care DBC[a]	783	24%	514	16%	561	17%	951	30%	717	22%	503	16%	509	16%	897	28%	716	22%	486	15%	501	16%	850	26%
Amount of standard outpatient care per patient (average costs in euros)	€ 638		€ 436		€ 477		€ 781		€ 586		€ 452		€ 420		€ 688		€ 572		€ 397		€ 419		€ 640	
Amount of medication for BD (average number of DDD[b])	128		133		130		132		126		133		129		130		127		129		127		130	
**Crisis psychiatric care**																								
All patients	665	21%	616	19%	568	18%	566	18%	508	16%	497	15%	486	15%	502	16%	471	15%	442	14%	443	14%	445	14%
Patients receiving standard outpatient care with medication for BD	218	10%	199	9%	189	9%	234	11%	168	8%	157	7%	152	7%	196	9%	152	7%	123	6%	139	7%	150	7%
Patients receiving standard outpatient care without medication for BD	47	11%	36	9%	29	8%	48	13%	25	7%	30	9%	23	7%	23	7%	33	10%	21	6%	19	6%	32	10%
Patients receiving only medication for BD	309	86%	308	80%	278	75%	223	68%	261	66%	252	65%	252	62%	232	59%	226	57%	251	57%	232	55%	214	50%
Patients without standard outpatient care	91	47%	73	32%	72	27%	61	21%	54	17%	58	17%	59	16%	51	14%	60	15%	47	12%	53	13%	49	12%
Subgroup with continuity of standard outpatient care[c]	348	17%	317	16%	284	14%	284	14%	234	12%	227	11%	236	12%	233	12%	229	11%	207	10%	212	11%	216	11%
Subgroup with quarters without standard outpatient care	317	26%	299	25%	284	23%	282	23%	274	23%	270	22%	250	21%	269	22%	258	21%	235	19%	231	19%	229	19%
Number of patients starting a new crisis psychiatric care DBC	206	6%	144	4%	131	4%	220	7%	170	5%	156	5%	156	5%	176	5%	155	5%	120	4%	118	4%	162	5%
Amount of crisis psychiatric care per patient (average costs in euros)	€ 1477		€ 1163		€ 1069		€ 1665		€ 1214		€ 1208		€ 1126		€ 1354		€ 1251		€ 880		€ 848		€ 1341	
**Somatic care**																								
Average adjusted costs of somatic care (euros)	€ 565		€ 635		€ 622		€ 645		€ 612		€ 600		€ 660		€ 664		€ 681		€ 690		€ 657		€ 653	
**Co-payments**																								
Co-payments for all care under the health insurance law (per year in euros)	€ 155								€ 165								€ 170							
Co-payments for psychiatric outpatient and inpatient care																								
**Price and cost indices for psychiatric and somatic care**																								
Price index psychiatric care (treatment)	100								98								99							
Price index psychiatric care (hospital bed)	100								98								99							
Cost index somatic care	100		96		94		98		100		97		96		101		105		100		98		102	

[a] DBC: Diagnoses Treatment Combination

[b] DDD: Defined Daily Dose

c Continuity of standard outpatient care was defined as receiving standard outpatient care during every quarter of 2009-2014.

[d] Results of testing deviations of trends with ARIMA are indicated in the tabel: [grey box] means downwards level shift [white box] means upwards level shift ____ means additve outlier

Table 1 Continued

Year	2012								2013								2014							
Quarter	1		2		3		4		1		2		3		4		1		2		3		4	
	N	%	N	%	N	%	N	%	N	%	N	%	N	%	N	%	N	%	N	%	N	%	N	%
**Standard outpatient care**																								
Patients receiving standard outpatient care with medication for BD	1962	61%	1895	59%	1917	60%	1903	59%	1871	58%	1890	59%	1883	59%	1910	60%	1842	57%	1847	58%	1823	57%	1847	58%
Patients receiving standard outpatient care without medication for BD	301	9%	293	9%	233	7%	226	7%	247	8%	228	7%	240	7%	202	6%	268	8%	247	8%	268	8%	228	7%
Patients receiving only medication for BD	500	16%	549	17%	545	17%	570	18%	556	17%	565	18%	555	17%	552	17%	583	18%	587	18%	587	18%	586	18%
Patients without standard outpatient care	447	14%	473	15%	515	16%	511	16%	536	17%	527	16%	532	17%	546	17%	517	16%	529	16%	532	17%	549	17%
All Patients receiving standard outpatient care	2263	70%	2188	68%	2150	67%	2129	66%	2118	66%	2118	66%	2123	66%	2112	66%	2110	66%	2094	65%	2091	65%	2075	65%
All Patients receiving medication for BD	2462	77%	2444	76%	2462	77%	2473	77%	2427	76%	2455	76%	2438	76%	2462	77%	2425	76%	2434	76%	2410	75%	2433	76%
Lithium	1252	39%	1248	39%	1231	38%	1241	39%	1185	37%	1202	37%	1214	38%	1220	38%	1194	37%	1179	37%	1161	36%	1160	36%
Antipsychotic medication	1420	44%	1406	44%	1422	44%	1443	45%	1423	44%	1435	45%	1423	44%	1449	45%	1439	45%	1441	45%	1419	44%	1454	45%
Anticonvulsant medication	938	29%	949	30%	939	29%	942	29%	946	29%	941	29%	951	30%	976	30%	954	30%	964	30%	954	30%	961	30%
Antidepressant medication	927	29%	918	29%	922	29%	932	29%	907	28%	933	29%	922	29%	946	29%	939	29%	937	29%	933	29%	925	29%
No bipolar disorder medication	748	23%	766	24%	748	23%	737	23%	783	24%	755	24%	772	24%	748	23%	785	24%	776	24%	800	25%	777	24%
Number of patients starting a new standard outpatient care DBC	637	20%	389	12%	485	15%	769	24%	576	18%	401	12%	488	15%	708	22%	624	19%	395	12%	468	15%	638	20%
Amount of standard outpatient care per patient (average costs in euros)	€ 514		€ 331		€ 428		€ 645		€ 458		€ 365		€ 429		€ 579		€ 503		€ 357		€ 406		€ 524	
Amount of medication for BD (average number of DDD)	126		126		126		129		121		124		125		129		124		127		125		128	
**Crisis psychiatric care**																								
All patients	428	13%	422	13%	440	14%	448	14%	440	14%	423	13%	427	13%	422	13%	391	12%	380	12%	384	12%	395	12%
Patients receiving standard outpatient care with medication for BD	124	6%	111	6%	142	7%	175	9%	163	9%	148	8%	156	8%	168	9%	142	8%	139	8%	153	8%	159	9%
Patients receiving standard outpatient care without medication for BD	20	7%	23	8%	18	8%	17	8%	23	9%	23	10%	31	13%	20	10%	23	9%	20	8%	26	10%	27	12%
Patients receiving only medication for BD	236	47%	238	43%	225	41%	215	38%	211	38%	212	38%	202	36%	195	35%	199	34%	195	33%	176	30%	178	30%
Patients without standard outpatient care	48	11%	50	11%	55	11%	41	8%	43	8%	40	8%	38	7%	39	7%	27	5%	26	5%	29	5%	31	6%
Subgroup with continuity of standard outpatient care	200	10%	198	10%	210	11%	231	12%	229	11%	219	11%	219	11%	220	11%	201	10%	192	10%	207	10%	214	11%
Subgroup with quarters without standard outpatient care	228	19%	224	18%	230	19%	217	18%	211	17%	204	17%	208	17%	202	17%	190	16%	188	16%	177	15%	181	15%
Number of patients starting a new crisis psychiatric care DBC	131	4%	124	4%	127	4%	153	5%	162	5%	151	5%	178	6%	178	6%	139	4%	146	5%	149	5%	170	5%
Amount of crisis psychiatric care per patient (average costs in euros)	€ 1217		€ 1122		€ 1129		€ 1328		€ 964		€ 878		€ 1280		€ 1236		€ 774		€ 1007		€ 1085		€ 1480	
**Somatic care**																								
Average adjusted costs of somatic care (euros)	€ 702		€ 748		€ 744		€ 754		€ 866		€ 882		€ 865		€ 941		€ 870		€ 853		€ 876		€ 937	
**Co-payments**																								
Co-payments for all care under the health insurance law (per year in euros)	€ 220								€ 350								€ 360							
Co-payments for psychiatric outpatient and inpatient care	*Psychiatric co-payments*																							
**Price and cost indices for psychiatric and somatic care**																								
Price index psychiatric care (treatment)	100								104								115							
Price index psychiatric care (hospital bed)	83								85								86							
Cost index somatic care	102		105		102		108		109		108		106		111		110		110		109		113	

Table 1 Continued

Year	2009				2010				2011			
Quarter	1	2	3	4	1	2	3	4	1	2	3	4
**Deviations in Trends: p-values, level shifts and additive outliers[d]**												
**Standard outpatient care**												
Patients receiving outpatient psychiatric care and bipolar disorder medication												
Patients receiving outpatient psychiatric care without bipolar disorder medication									0,0018			
Patients receiving only bipolar disorder medication									0,0241			
Patients without standard outpatient care												
All Patients receiving standard outpatient care												
All Patients receiving bipolar disorder medication								0,0002				
Lithium												
Antipsychotic medication												
Anticonvulsant medication												
Antidepressant medication												
No bipolar disorder medication												
Number of patients starting a new standard outpatient DBC treatment												
Amount of outpatient psychiatric care per patient (average costs in euros)												
Amount of bipolar disorder medication (average number of DDD)												
**Crisis psychiatric care**												
All patients												
Patients receiving outpatient psychiatric care and bipolar disorder medication												
Patients receiving outpatient psychiatric care without bipolar disorder medication												
Patients receiving only bipolar disorder medication												
Patients without standard outpatient care												
Subgroup with continuity of standard outpatient care												
Subgroup with quarters without standard outpatient care												
number of patients starting a new crisis psychiatric DBC treatment												
Amount of crisis psychiatric care per patient (average costs in euros)												
**Somatic care**												
Average adjusted costs of somatic care (euros)												

Table 1 Continued

Year	2012				2013				2014			
Quarter	1	2	3	4	1	2	3	4	1	2	3	4
**Deviations in Trends: p-values, level shifts and additive outliers**												
**Standard outpatient care**												
Patients receiving standard outpatient care with medication for BD	0.0001				0.0026	< 0.0001						
Patients receiving standard outpatient care without medication for BD			0.0014				< 0.0001					
Patients receiving only medication for BD	0.0008			0.0609		0.0003						
Patients without standard outpatient care						< 0.0001						
All Patients receiving standard outpatient care		0.0017				< 0.0001						
All Patients receiving medication for BD			< 0.0001									
Lithium												
Antipsychotic medication												
Anticonvulsant medication												
Antidepressant medication												
No bipolar disorder medication												
Number of patients starting a new standard outpatient care DBC	0.0020		0.0172			0.0426			0.0448			
Amount of standard outpatient care per patient (average costs in euros)												
Amount of medication for BD (average number of DDD)												
**Crisis psychiatric care**												
All patients		0.0010										
Patients receiving standard outpatient care with medication for BD		< 0.0001			0.0265			0.0071				
Patients receiving standard outpatient care without medication for BD												
Patients receiving only medication for BD	0.0040											
Patients without standard outpatient care												
Subgroup with continuity of standard outpatient care			0.0018									
Subgroup with quarters without standard outpatient care		0.0033	0.0344									
Number of patients starting a new crisis psychiatric care DBC		0.0046										
Amount of crisis psychiatric care per patient (average costs in euros)												
**Somatic care**												
Average adjusted costs of somatic care (euros)		0.0314										

Table 2 Trends of psychiatric and somatic care and general co-payments, parameters of time series analysis (ARIMA)

	parameters time-series analysis (ARIMA)[a]			
	AIC	mu	ma1	
		p-value	p-value	
**Standard outpatient care**				
Patients receiving standard outpatient care with medication for BD	-149	< 0.0001	0.0007	
Patients receiving standard outpatient care without medication for BD	-146	< 0.0001	0.0412	
Patients receiving only medication for BD	-143	< 0.0001	0.0025	
Patients without standard outpatient care	-138	< 0.0001	0.0032	
All Patients receiving standard outpatient care	-133	< 0.0001	0.0006	
All Patients receiving medication for BD	-169	< 0.0001	0.0051	
Lithium				not tested
Antipsychotic medication				not tested
Anticonvulsant medication				not tested
Antidepressant medication				not tested
No medication for BD				not tested
Number of patients starting a new standard outpatient care DBC[b]	193	0.0109	0.0278	
Amount of standard outpatient care per patient (average costs in euros)				no deviations
Amount of medication for BD (average number of DDD[c])				no deviations
**Crisis psychiatric care**				
All patients	-127	< 0.0001	0.0661	
Patients receiving standard outpatient care with medication for BD	-146	< 0.0001	0.0194	
Patients receiving standard outpatient care without medication for BD				
Patients receiving only medication for BD	-71	< 0.0001	0.0485	
Patients without standard outpatient care				no deviations
Subgroup with continuity of standard outpatient care[d]	-112	< 0.0001	0.0023	
Subgroup with quarters without standard outpatient care	-144	< 0.0001	0.0098	
Number of patients starting a new crisis psychiatric care DBC	182	0.0030	0.0404	
Amount of crisis psychiatric care per patient (average costs in euros)				no deviations
**Somatic care**				
Average adjusted costs of somatic care (euros)	216	0.0018	0.0033	

[a] AIC: Akaike Information Criterium, mu: mean estimate, ma1: moving average estimate

[b] DBC: Diagnoses Treatment Combination

[c] DDD: Defined Daily Dose

[d] Continuity of standard outpatient care was defined as receiving standard outpatient care during every quarter of 2009-2014.
